# Effects of a Family Caregiver Care Programme in Musculoskeletal Pain and Disability in the Shoulder-Neck Region—A Randomised Clinical Trial

**DOI:** 10.3390/ijerph20010376

**Published:** 2022-12-26

**Authors:** Rocío Llamas-Ramos, Laura Barrero-Santiago, Inés Llamas-Ramos, Federico Montero-Cuadrado

**Affiliations:** 1 Department of Nursing and Physiotherapy, Faculty of Nursing and Physiotherapy, Universidad de Salamanca, Avda. Donantes de Sangre s/n, 37007 Salamanca, Spain; 2 Department of Cell Biology, Genetics, Histology and Pharmacology, Faculty of Medicine, University of Valladolid, Avda. Ramón y Cajal 7, 47005 Valladolid, Spain; 3 University Hospital of Salamanca, P.º de San Vicente, 182, 37007 Salamanca, Spain; 4 Unit for Active Coping Strategies for Pain in Primary Care, East-Valladolid Primary Care Management, Castilla and Leon Public Health System (Sacyl), 47011 Valladolid, Spain

**Keywords:** caregivers, exercise programme, musculoskeletal pain, neck pain, quality of life

## Abstract

Life expectancy in Spain has increased and older people need more health care to improve their quality of life. The high demands of the population sometimes collapse health services, making them insufficient to cover their needs, which leads to the development of “non-professional caregivers”. These caregivers have suffered musculoskeletal injuries of the cervical spine and shoulders and should be guided and assessed on ergonomics, biomechanics, or kinesiotherapy. However, there are no interventions to help them care for themselves. This study presents the application of a physical exercise programme to prevent these disorders in women caregivers of dependent patients. It consists of a randomised controlled clinical trial with two groups: both received a family caregiver care programme, and the intervention group also received a physical therapeutic exercise programme for 12 weeks. A total of 62 caregivers completed the study, who were mostly daughters or wives of dependents. Of these, 70.97% presented a “moderate” pain intensity and a cervical disability of 13.72 ± 7.64 points in the NDI questionnaire in the initial evaluation. In the intervention group of caregivers, there was a significant decrease in the pain intensity and a significant increase (*p* < 0.05) in all cervical joint amplitude movements. Caregivers present a high prevalence of musculoskeletal disorders. The physical exercise programme was effective in reducing the intensity of pain, lumbar disability, and cervical disability of the family caregivers.

## 1. Introduction

Life expectancy in Spain has increased in recent years, as has the percentage of older people who need more health care to improve their quality of life [1]. Health services sometimes collapse due to the high demands of the population or the shortage of health professionals, making them insufficient to cover the needs of the population, which gives rise to “non-professional caregivers” [2]. These caregivers often suffer from musculoskeletal disorders and pain; they should be guided and assessed on ergonomics, biomechanics, or kinesiotherapy by health services, but generally receive little information. [3].

Providing care or caregiving involves “assisting another person to perform activities which are necessary for survival, human functioning or social participation, or performing such activities for a person who is unable to do them” [4]. Depending on the level of dependency, the caregiver will have a greater or lesser physical workload [5].

The majority of caregivers are women (mostly older women), accounting for 84% of the total, and usually relatives of the patients, namely, wives and daughters [6,7,8,9]. Perhaps because of the existing social conception that attributes the role of caregiver to the female personality [10], this condition makes research necessary in this type of population [6]. This aspect should be considered, as it has been shown that women present with musculoskeletal pain in the following situations: they “experience higher pain intensity, greater pain-related interference with function, and more disability”. Women have also reported worse depression, anxiety, and self-efficacy [11], and these sex differences are generated by biological and biomechanical differences [12]. Furthermore, in relation to age, bone mineral density decreases with age, especially in women, being more evident after menopause when their musculoskeletal system becomes more susceptible to fractures [13].

These caregivers not only suffer from psychological stress [14,15] due to imbalances between demands and lack of resources available but also musculoskeletal disorders caused by the overloading of their tasks. Among their tasks as caregivers of dependent people with limited functional capacities are changes in decubitus, transfers, bathing, and changing diapers [6]. With the increase in time spent, caregivers have reported an increase in musculoskeletal disorders as well as high levels of pain [16]. This was also postulated by Darragh et al. in their study where they concluded that daily activities of care for dependent persons aggravated musculoskeletal pain [17].

Musculoskeletal disorders are a major health problem in developed countries because of their high prevalence [18,19]. They are defined as inflammatory and degenerative conditions affecting muscles, tendons, ligaments, joints, intervertebral discs, cartilage, bones, and peripheral nerves [20,21,22], and this pathology entails medical costs, work disability, and a decrease in the quality of life of patients who suffer from it [23,24].

Regarding caregivers, most of them have some active or passive restriction of movement affecting their musculoskeletal systems [5]. Specifically, in this population of women caregivers, musculoskeletal disorders are very frequent at the cervical spine [25]. The literature is scarce, but some authors have reported a prevalence of 38–60% [26,27], with the neck and shoulders being the most prevalent regions in a study published by Chang et al., in which they hypothesized that incorrect use of the upper arms rather than the trunk for moving or transferring patients would lead to the development of neck and shoulder pain [25]. Common risk factors include physical tasks such as repetitive lifting, handling, and transferring of patients, persistent awkward postures, and insufficient knowledge of body mechanics or transfer techniques [28,29,30]. However, there is little information in the literature regarding the physical demands of caregivers and their relation or possible association with injury [17].

Authors highlight the importance of information and education on risk factors associated with musculoskeletal disorders as well as psychological stress to reduce and prevent them, improving the quality of life of caregivers [3,25,31,32]. It has been agreed that prevention of musculoskeletal pain should be applied at the onset of pain [19,33]; programmes designed or created to prevent overload and even emotional impact that may affect the caregiver’s health and quality of life should therefore be investigated [34].

In this line of research, several interventions have been developed for the prevention, education, and self-care of these caregivers of dependent patients. One of the strategies has been “Safe Patient Handling and Mobility”. In this strategy, workers used equipment to lift and move patients, demonstrating a reduction in biomechanical loads, overexertion injuries, and working time [35,36,37]. However, these interventions are indicated for health professionals, caregivers in institutions or at home who have access to this equipment. Another line of research also aimed at this population of women caregivers has been telephone interventions with training courses, social intervention programmes, and medical and psychological care [38]. Finally, a physical exercise programme has been proposed from primary care physiotherapy on health-related quality of life, musculoskeletal pain, and healthy physical condition in family caregivers of dependent patients. An improvement in quality of life, and a decrease in subjective burden, anxiety, and depression have been achieved [39].

The interventions are aimed at treatments improving psychological well-being and quality of life but scant literature reflects the musculoskeletal aspect closely linked to quality of life. This study presents the application of a physical exercise programme aimed at preventing musculoskeletal injuries of the cervical spine and shoulders in women caregivers of dependent patients.

## 2. Materials and Methods

### 2.1. Study Design

A randomised controlled clinical trial was performed between February and May 2017. The study was implemented in Valladolid in two medical centers (Pilarica and Circular PC), and the sample was recruited in the study entitled Effectiveness of a Physical Therapeutic Exercise Programme for Caregivers of Dependent Patients: A Pragmatic Randomised Controlled Trial from Spanish Primary Care [39]. The study was approved by the Ethical Committee for Clinical Research (CEIC) of Valladolid-East Health Area (CEIC: PI-13-88) before the onset of the study, and was registered at ClinicalTrials.gov with the identifying code NTC03675217. The trial was conducted in accordance with the recommendations of the SPIRIT statement [17] and CONSORT statement [40]. In addition, the PRECIS-2 tool was used for the study design [41].

### 2.2. Participants

Female family caregivers belonged to two public centers of the Valladolid-East Health Area of SACYL (public service of Health of Castile y Leon, Spanish Public Health System) participated in the study. A random alphanumeric code was assigned to each participant to establish both groups. In addition, the research group who implemented the evaluations were blinded to the patients’ allocation. The inclusion criteria were: to be female, included in the family caregiver care programme (FCCP) from the two participant centers, aged more than 18 years, to have been the main caregiver for at least 6 months, to have had no changes in medication for at least 3 months prior to assessment, and having signed the informed consent. The exclusion criteria were: having associated pathologies that could prevent caregivers from performing the physical exercise programme (moderate-intensity exercise), participation in another family care programme for caregivers, participation in any regular physical activity programme, performing regular physical activity (3 days a week at least), losing the caregiver condition (moving to another town, death, or institutionalization of the relative), and having support from one or more full-time formal caregivers to care for the dependent relatives.

To estimate the power of the sample, we considered the differences of two points when testing the equality of SF-36 summary of physical health means [42,43,44], with a type I error of 5%. Including 68 participants, the estimated power was 80%.

### 2.3. Variables and Measurement Instruments

Data were collected following a standardized protocol [39].

#### 2.3.1. Musculoskeletal Pain

Pain intensity and location were evaluated.

-Visual Analogue Scale was employed to measure pain intensity. It consisted of a line, vertical or horizontal, continuous 100 mm without any inscription or representation, except at the ends, where the inscription “no pain” appeared on the far left (measured as 0) and on the far right, “worst pain imaginable” [45]. To accept a relevant clinical improvement, there must have been a difference of 10 to 20 mm in the VAS assessment range [46]. Participants implemented themselves on the scale with the pain felt during the previous week of the evaluation.

-Body maps were employed to measure pain location. McGill questionnaire body maps were used. They were of a two-dimensionally represented human figure with four parts that corresponded to the anterior, posterior, left, and right side of the body [47]. Participants implemented themselves on the scale with the pain location during the previous week of the evaluation.

#### 2.3.2. Cervical Disability

-Neck Disability Index was employed to measure cervical disability. It was made up of 10 sections; four were related to subjective symptoms (headache, dreams, intensity of pain, and ability to concentrate), and the remaining six with activities of daily living (personal care, lifting weights, reading, work, driving, and leisure activities). Each of the sections offered six possible answers. The patient was obliged to choose the item that best described the situation at the current time, scoring each of them from 0 to 5 depending on the answer. The total score range was from 0 to 50 points, so that greater disability was presented the closer it was to 50 points (314). Regarding the punctuation, it was established that a score less than 9% indicated “no disability”; between 10 and 29% indicated “slight disability”; between 30–49%, “moderate disability”; between 50–69%, “severe disability”, and scores greater than 70% represented a “complete disability” [48].

#### 2.3.3. Range of Motion (ROM)

Range of motion of shoulders and cervical spine were evaluated.

-Shoulders. A digital goniometer Axis-TM^®^ was employed. Flexion, extension, and abduction of both shoulders were evaluated following the Taboadela protocol [49].

-Cervical spine. A CROM Basic goniometer (Cervical Range of Motion Instrument) was selected to measure the range of motion in the three planes of space: sagittal plane (flexo-extension), frontal plane (side-bending) and transverse plane (rotations).

#### 2.3.4. Strength—Muscular Endurance

-Manual dynamometry was performed with a digital dynamometer TKK-5401 model. Two attempts were made with each hand alternately, with a minimum rest of one minute between them. Each attempt lasted 3 s [50].

### 2.4. Procedure

The research team performed the evaluations. The evaluations consisted of self-administered questionnaires and a functional test battery [39]. Participants were randomly assigned into two groups: control group and intervention group. Every participant was evaluated twice, before and after the intervention.

Both groups received the family FCCP and the intervention group also received a physical therapeutic exercise programme. The FCCP performed 4 sessions and was carried out by a nurse (1st and 2nd session), a social worker (3rd session), and a physiotherapist (4th session). These professionals implemented the programme without knowing which group each of the family caregivers belonged to (blinded). The intervention group performed a physical therapeutic exercise programme for 12 weeks. Each session lasted 60 min with a frequency of 3 sessions per week (alternate days), for a total of 36 sessions. The programme was carried out as a community (in groups of 15–20 caregivers) and at different times to make it easier for caregivers to attend in the best conditions. The sessions were structured in three phases: warm-up phase (10–15 min), main part phase (35–40 min), and cooling-down phase (10–15 min) [39].

### 2.5. Statistical Analysis

Quantitative variables were summarised with means and standard deviations, and qualitative variables were summarised with percentages. We calculated 95% confidence intervals (CI95%) for means and percentages. The Kolmogorov–Smirnov test showed a normal distribution of the data, and the Student’s t-test to test changes in numerical variables within each treatment group and between groups was used.

The effect size was calculated, corresponding to the comparison of the intervention of the IG with the CG, using Cohen’s “d” statistic. A *p*-value of less than 0.05 was considered statistically significant. Statistical analyses were carried out using the R v3.2.3 statistical package (R Foundation for Statistical Computing, Vienna, Austria).

## 3. Results

### 3.1. Sample Characteristics

A total of 62 caregivers completed the study. The mean age of the caregivers was 64.35 ± 7.56 years, with the youngest caregiver being 47 years old and the oldest caregiver 76 years old. The highest percentage of the participants (53.22%) were between 59 and 70 years old.

Regarding the marital status situation, 66.12% were married and 16.12% were single. A total of 64.51% of the caregivers had primary studies, although it should be noted that 8.06% of them did not have any type of academic training. As regards employment, most of the caregivers were not working at the time of the investigation; 40.32% of them were retired and 27.41% had never worked.

No statistically significant differences (*p* > 0.05) were found in the sociodemographic variables of the caregivers between the two research groups. (Table 1).

Regarding the care situation, Table 1 highlights that the caregivers were mostly daughters (48.38%) or wives (37.09%) of dependents.

All the caregivers in the study lived with their dependent family member. A total of 41.93% of the caregivers received help from a cohabiting relative, while 25.80% did not have any type of help for the care of the dependent relative.

None of the caregivers declared receiving help from a neighbour, friend and/or volunteer. In relation to the hours of care provided by the caregivers to their dependent family members, it stands out that 72.58% of the caregivers dedicated more than 12 h a day to the care and needs of their dependent family member. Lastly, it should be noted that, on average, the caregivers had been caring for their family member for 74.65 ± 59.45 months.

There were no statistically significant differences (*p* > 0.05) between the groups in any of these commented variables (Table 1).

### 3.2. Muskuloskeletal Pain

Most of the caregivers in the sample (70.97%) presented a “moderate” pain intensity in the initial evaluation, according to the VAS scale.

Table 2 describes the baseline MSD characteristics of the caregivers in the sample. The caregivers in the study reported MSD in the week prior to the initial evaluation, presenting a mean intensity of pain according to the VAS scale of 6.20 ± 1.18 cm. The first episode of MSD, according to the clinical history, had occurred 45.26 ± 26.85 months on average before starting the study. Regarding the number of locations with pain, the mean was 2.58 ± 1.19 body areas with MSD, being the minimum one and the maximum six, out of a maximum of seven possible locations. No statistically significant differences were found between the groups (*p* > 0.05) in any of the variables mentioned in the pre-intervention evaluation.

The pre-post-intervention assessment of caregivers’ MSD intensity showed that in the CG, there was an increase in the mean score (VAS scale), but this difference wasn’t statistically significant (*p* > 0.05). On the other hand, in the IG there was a significant decrease in the intensity of MSD (*p* < 0.001). It is noteworthy that after the physical exercise programme, all the IG caregivers who had presented pain intensity above 70 mm on the VAS scale (28.12%) managed to reduce their pain intensity to below this level (severe pain). In the intergroup analysis, statistically significant differences also occurred in favour of IG with a large effect size (d= −2.66) (Table 3).

### 3.3. Cervical Disability

The caregivers of the sample presented in the initial evaluation a cervical disability of 13.72 ± 7.64 points in the NDI questionnaire. Only 11.29% of the caregivers did not present cervical disability, with most of them presenting mild disability (46.77%) and moderate disability (33.87%). None of the caregivers presented complete cervical disability according to the questionnaire.

Regarding the effects of the intervention on cervical disability in the CG, there was a significant increase in cervical disability (*p* < 0.05), in contrast to the IG where there was also a significant decrease (*p* < 0.001). Furthermore, in the IG, 25% of caregivers who initially presented some degree of cervical disability in the initial assessment, did not present any degree of cervical disability in the post-intervention assessment. In the intergroup comparison, the differences were statistically significant, and the effect size was also large (d= −2.49) in favour of the IG (Table 3).

### 3.4. Range of Motion

The total sample of caregivers initially presented a mean cervical flexion-extension mobility of 84.69 ± 7.77 degrees, mean lateral flexion 61.25 ± 9.72 degrees, and a mean global rotation of 114.19 ± 19.20. No statistically significant differences were found between groups (*p* > 0.05) in any of these variables (Table 2).

After the programme, cervical ROM obtained no significant changes in the CG except in the extension movement, which underwent a significant reduction after the intervention (*p* < 0.05). In the IG caregivers, there were significant increases (*p* < 0.05) in all cervical joint amplitude movements, except for the global lateral flexion movement, in which there was an increase but it was not statistically significant (*p* > 0.05). The same occurred in the intergroup comparison; significant differences (*p* < 0.05) were found between groups in all cervical joint amplitude movements except global lateral flexion movement. The effect size was small for lateral flexion (d = 0.34), moderate for flexion (d = 0.51), and large for all other cervical movements (Table 3).

Considering the shoulder joint range of motion (ROM), the caregivers obtained a mean flexion mobility of 155.16 ± 5.26 degrees in the right shoulder and 152.5 ± 13.49 degrees in the left shoulder. The extension movement obtained a mean of 31.13 ± 6.87 degrees for the right shoulder and 30.34 ± 7.07 for the right. Finally, the separation was 152.61 ± 16.41 degrees on the right shoulder and 149.29 ± 15.91 degrees on average on the caregivers’ left shoulder. No significant differences were found between groups (*p* > 0.05) in any of the commented variables (Table 2).

After the intervention, in the shoulder ROM there were significant increases (*p* < 0.005) in the degrees of all the movements assessed in the IG caregivers. In contrast, no changes were observed in the CG, except in left shoulder abduction where there was a significant decrease (*p* < 0.05) (Table 3). Statistically significant differences were found between groups in favour of the IG, in all shoulder movements of the caregivers assessed, except for the left shoulder extension movement, with a large effect size in all of them (Table 3).

### 3.5. Strength—Muscular Endurance

The family caregivers in the sample initially presented, in the manual dynamometry test, mean values of maximum bimanual grip strength (right hand grip strength + left hand grip strength) of 40.66 ± 7.09 kg (Table 2). The mean maximum hand grip strength tended to be higher in the right hand (dominant hand in almost all caregivers) than in the left hand, without these differences being significant.

There were no significant differences between groups (CG versus IG) (Table 2).

In relation to the maximal hand grip strength, after the intervention, there was a decrease in it in the CG caregivers, while in the IG it increased, with both differences being statistically significant (*p* < 0.05). In the intergroup comparison, statistically significant differences were also found (*p* < 0.001), and with a large effect size (d = 2.23) (Table 3).

## 4. Discussion

Musculoskeletal pain has a high prevalence in the general population [51]. However, there are few studies on the prevalence of neck pain in Spain, such as those published by Catalá et al. and Fernández de las Peñas et al., which have reflected a prevalence of 21.5% of back pain in general [52], while specifically for neck pain, it has been estimated at 19.5% [53] without there being numerical data of evidence available for caregivers of dependent people. These data are lower than our findings where 70.97% of the sample experienced moderate pain. This neck pain is more prevalent in women, which were the target of our study, and is associated with a worse state of health, being a significant burden for people and for the country’s economy [53,54,55], with high costs at a European level [56] and reaching up to $635 billion a year in the US [57].

In the working population, specifically in nurses, musculoskeletal pain has been extensively investigated [37,58,59,60], with this population presenting up to 75% of symptoms of musculoskeletal pain [61]. However, for family caregivers there is little evidence. As it has been stated previously, patient lifting and transferring are postulated as the main risk factors for this pathology [62,63]; for this reason, several treatment interventions have been carried out, and it has been verified that neck/shoulder pain was greatly reduced thanks to the interventions of the “Safe Handling and Mobility” programme, where the study participants demonstrated a decrease in pain, aching, stiffness, burning, numbness, or tingling in the lower back, neck, shoulders, or hands/wrists in the past 12 months [37].

Despite not being a recognized job to be able to establish comparisons with these articles, caregivers also perform physical work with repetitive movements, the frequency and duration of these transfers being related to the increased risk of injuries [17,64]. Interventions to reduce such physical effort, such as use of patient handling slings during transfers (sit-to-stand or stand pivot), mechanical lifts, or educational programmes are necessary for prevention, thus reducing the risk of injury [65]. It is important to highlight that caregivers of dependent people complain of moderate to chronic spinal pain, with the cervical region being the most prevalent, reporting symptoms such as movement dysfunction, swelling, numbness, weakness, and tiredness [5], including back pain and headache [66]; they have even expressed that the symptoms worsen with caregiving [17]. It should be noted that caregivers are also more likely to develop stress [67]. Many of them self-medicate with analgesics, anti-inflammatories, or antibiotics; this was revealed in a study carried out with 92 caregivers who reflected the taking of medication as a consequence of caring for family members at home [68]. Taking into account these risk factors and high prevalence, this programme has been developed and tested to reduce their discomfort and to improve their quality of life.

Research on non-professional caregivers (family and unpaid) is booming, and the negative impact of this care on the well-being and physical and mental health of caregivers is evident, which implies a personal, social, and economic cost that must be considered [69,70], since they sometimes refer to a feeling of discontent and abandonment by the health services. This population represents a key element in the maintenance and care of elderly and disabled or dependent people [71]. In addition, the care that this population offers prevents the institutionalization of patients and reduces hospital stays [72].

Musculoskeletal problems in caregivers of children with cerebral palsy [73,74,75,76,77,78], stroke [70,71,79,80], in multiple sclerosis [81], or in the elderly [71,82,83], have been investigated in several studies, demonstrating physical overload as the main cause of risk for these ailments in their study with a sample of almost 400 caregivers (mothers and fathers) [84], establishing a prevalence of 35.7%. This was only surpassed by low back pain [73], which has been linked to higher levels of depression and poorer quality of life. However, no studies related to female caregivers of dependent people have been found.

Most of the caregivers reflected in the literature are wives, this condition being an opportunity to keep the family together [9], which agrees with our study where, in 37.1% of the sample, the caregivers were the patient’s wives, being surpassed by daughters by 48.4%. Other studies have also shown that “informal care” is performed more frequently by women [85] and by adults between the ages of 50 and 70 [86]. In a study published by Marco et al., they investigated the differences between men and women aged 45 to 65 years, without finding differences in the perception of health of the former, while women referred to diminished quality of life values in relation to the general population [71].

In addition, the mean age of our sample of caregivers was 64.35 ± 7.56 years, which is consistent with published studies where the sample of caregivers was between 25 and 60 years old, and showed the most frequent osteomuscular problems in the lumbar region, lower limbs, shoulders, cervical region, arms and dorsal region [6,87]. A slightly wider range was reflected in the study by Leite et al. [68], where the age range varied from 58 to 97 years; Faronbi et al. included caregivers over 60 years of age [83], while for others it was more common to be over 75 years of age [88]. In the present study, the age range varied from 47 to 76 years with moderate localized pain in at least two body regions, the most prevalent being the lumbar region (69.4%) and the cervical region (56.7%).

The importance of the care and attention of caregivers lies in the fact that the ailments or disabilities they present reflect what they have experienced during the process of caring for their relatives to the detriment of their own health, and may generate, apart from neck pain, weight loss, muscle contractions, tachycardia, and even asthenia [6]. Other authors have postulated that the presence of depression and musculoskeletal pain in the caregiver, the time spent caring for the patient, and functional motor dependence are related to a greater deterioration in the health-related quality of life of the caregivers [71]. A positive relationship has been shown between the time dedicated to caring for patients and the level of effort presented by the caregiver [71], sometimes exceeding 47 h a week [88].

Specifically in the present study, 72.58% of the caregivers reflected dedicating more than 12 h a day to the care of the relative, without any type of external help, representing a cervical disability of 113.72 ± 7.64 points in the NDI questionnaire, being mild disability in 46.77% and moderate in 33.867%. In addition, it should be noted that, considering the average age of the caregivers, it seems evident that most of them have associated physical limitations, such as fatigue or lack of energy, including physical inactivity, which will also interfere with their quality of life [83].

Along this line, an article was published that investigated this asthenia or feeling of fatigue that caregivers presented, showing results of triggers of mental fatigue, lack of concentration, apathy, anxiety, depression, and muscle fatigue [89]. This lack of care reflects injuries in caregivers of dependent people with limited functional capacity, over 60 years of age, as stated by Costa et al. in their publication [89].

Interventions aimed at educating and caring for this population are very important, since most of their interventions are associated with the risk of developing some musculoskeletal ailment [90], again being more prevalent in women [91]. Some authors have highlighted education to mobilize patients [6] while others have developed a web-based daily training aimed at improving the quality of life and health of these caregivers, trying to reduce depression, anxiety, stress, and pain [78]. Most of these interventions are aimed at assessing the quality of life of caregivers, but the effectiveness of these interventions has not been considered [92,93,94], which is why Grant et al. conducted a telephone intervention programme to evaluate such interventions [38]. In the present study, we have focused on a 12-week exercise programme that consisted of three weekly sessions, performing a total of 36 sessions to work on mobility and muscle strength. The sessions were divided into an initial warm-up part, a central work part, and a relaxation and cool-down part. Within the mobility limitations for the cervical and shoulder region presented by these caregivers, a significant increase was observed in almost all the variables of the caregivers of the intervention group in relation to the control group, in the same way as for the evaluation of strength of the hand, differences were also found between the established groups, increasing in the experimental group and decreasing in the control group.

The main limitations of this study were that it was only carried out with women, which prevents the extrapolation of the data to other types of population, such as men, as well as the impossibility of guaranteeing double blinding, as the women knew which group they belonged to. Finally, the results were only taken into account in the short term; studies that assess the treatment effects in the long term should be considered in future work.

## 5. Conclusions

Non-professional caregivers of dependent patients suffer musculokeletal disorders and pain. The physical exercise programme discussed in this study was effective in reducing pain intensity, lumbar disability, and cervical disability of family caregivers of dependent patients. The physical exercise programme was effective in improving the healthy physical condition of the caregivers in all dimensions, thereby improving their quality of life.

## Figures and Tables

**Table 1:** Sample characteristics.

	Total Sample (*n* = 62)	Control Group (*n* = 30)	Intervention Group (*n* = 32)
Age (years) ^a^	64.35 ± 7.56	63.77 ± 7.83	64.91 ± 7.38
Age ^b^:			
<59 years	15 (24.19)	9 (30.00)	6 (18.75)
59–70 years	33 (53.22)	14 (46.66)	19 (59.37)
>70 years	14 (22.58)	7 (23.33)	7 (21.87)
Marital/Civil Status ^b^:			
Single	10 (16.12)	6 (20.00)	4 (12.5)
Married	41 (66.12)	18 (60.00)	23 (71.9)
Separated	6 (9.67)	4 (13.33)	2 (6.25)
Widowed	5 (8.06)	2 (6.66)	3 (9.37)
Study level ^b^:			
No studies	5 (8.06)	2 (6.66)	3 (9.37)
Primary	40 (64.51)	19 (63.33)	21 (65.62)
Secondary	8 (12.90)	5 (16.66)	3 (9.37)
University	9 (14.52)	4 (13.33)	5 (15.62)
Working ^b^:			
Retired	25 (40.32)	11 (36.66)	14 (43.75)
Employed	9 (14.52)	5 (16.66)	4 (12.50)
Unemployment	11 (17.74)	7(23.33)	4 (12.50)
Never worked	17 (27.41)	7 (23.33)	10 (31.25)
Kinship ^b^:			
Daughter	30 (48.38)	14 (46.66)	16 (50.00)
Spouse	23 (37.09)	12 (40.00)	11 (34.37)
Mother	3 (4.83)	1 (3.33)	2 (6.25)
Sister	3 (4.83)	2 (6.66)	1 (3.12)
Daughter-in-law	3 (4.83)	1 (3.33)	2 (6.25)
Help with care ^b^:			
No	16 (25.80)	7 (23.33)	9 (28.12)
Yes, cohabiting relative	26 (41.93)	12 (40.00)	14 (43.75)
Yes, non-cohabiting relative	11 (17.74)	6 (20.00)	5 (15.62)
Yes, paid person	9 (14.52)	5 (16.66)	4 (12.50)
Care Hours:			
1–6 h	2 (3.22)	1 (3.33)	1 (3.12)
6–12 h	15 (24.19)	8 (26.66)	7 (21.88)
>12 h	45 (72.58)	21 (70.00)	24 (75.00)
Months of care ^a^	74.65 ± 59.45	70.8 ± 68.13	78.5 ± 50.77
Years of care ^a^	5.35 ± 3.75	4.57 ± 3.08	6.09 ± 4.21

^a^ median ± standard deviation; ^b^ *n* (%).

**Table 2:** Initial results (media ± standard deviation) of pain intensity, cervical and lumbar disability, flexibility, and range of motion of family caregivers.

Variable	Total Sample (*n* = 62)	Control Group (*n* = 30)	Intervention Group (*n* = 32)
Pain Intensity (VAS)	6.20 ± 1.18	6.07 ± 1.28	6.32 ± 1.07
Cervical Disability (NDI)	13.72 ± 7.64	13.67 ± 8.36	13.78 ± 7.04
Cervical ROM (degrees):			
Flexo-Extension	84.69 ± 7.77	84.60 ± 8.04	84.78 ± 7.63
Flexion	36.66 ± 3.21	36.17 ± 3.53	37.13 ± 2.86
Extension	48.03 ± 6.22	48.43 ± 5.39	47.66 ± 6.97
Total Lateral Flexion	61.25 ± 9.72	62.30 ± 9.62	60.28 ± 9.86
Right Lateral Flexion	30.82 ± 5.07	30.07 ± 4.79	31.53 ± 5.31
Left Lateral Flexion	30.44 ± 5.82	32.23 ± 5.67	28.75 ± 5.53
Global Rotation	114.19 ± 19.20	117.01 ± 18.36	111.56 ± 19.88
Right Rotation	58.08 ± 9.94	59.1 ± 10.08	57.13 ± 9.87
Left Rotation	56.11 ± 10.47	57.9 ± 9.40	54.44 ± 11.26
Shoulder ROM (degrees):			
Right Flexion	155.16 ± 5.26	156.57 ± 15.75	153.84 ± 14.93
Left Flexion	152.5 ± 13.49	153.43 ± 15.03	151.63 ± 12.06
Right Extension	31.13 ± 6.87	32.3 ± 7.02	30.03 ± 6.64
Left Extension	30.34 ± 7.07	29 ± 7.48	31.59 ± 6.53
Right Abduction	152.61 ± 16.41	154.4 ± 16.67	150.94 ± 16.23
Left Abduction	149.29 ± 15.91	150.90 ± 15.93	147.78 ± 16.00
Handgrip Strength (kg/m^2^)	40.66 ± 7.09	41.43 ± 6.76	39.92 ± 7.42
Right Hand (kg/m^2^)	20.61 ± 3.73	20.98 ± 3.40	20.26 ± 4.04
Left Hand (kg/m^2^)	20.05 ± 3.57	20.47 ± 3.53	19.65 ± 3.61

Statistically significative differences between groups; *p* < 0.05.

**Table 3:** Intervention Results (media ± standard deviation) of pain intensity, lumbar and cervical disability, anterior trunk flexibility, shoulder and cervical ROM, maximal hand grip, and abdominal and lower limb strength-resistance (SR).

Dimension (Instrument)	Group CG (*n* = 30) IG (*n* = 32)	Pre	Post	Intra-Dif (IC 95%)	Inter-Dif (IC 95%)	ES
Pain Intensity (VAS)	CG	60.70 ± 12.80	63.01 ± 12.40	−2.3 (−0.54–0.09)	−25.7 (−30.6–−20.81) ‡	−2.66
	IG	63.20 ± 10.70	39.80 ± 12.80	23.4 (19.5–27.3) **		
Cervical Disability (NDI)	CG	13.67 ± 8.36	14.68 ± 8.10	−1.01 (−1.612–−0.38) *	−6.82 (−8.19–−5.43) ‡	−2.49
	IG	13.78 ± 7.04	7.97 ± 5.33	5.81 (4.55–7.07) **		
Cervical ROM(degrees)						
Flexo-Extension	CG (30)	84.6 ± 8.04	80.63 ± 8.95	3.97 (1.97–5.96) **	14.25 (10.61–17.89) ‡	1.98
	IG (32)	84.78 ± 7.63	95.06 ± 7.90	−10.28 (−13.40–−7.16) **		
Flexion	CG (30)	36.17 ± 3.53	35.87 ± 4.56	0.30 (−1.05–1.65)	2.14 (0.03–4.26) †	0.51
	IG (32)	37.13 ± 2.86	38.97 ± 4.60	−1.84 (−3.53–−0.16) *		
Extension	CG (30)	48.43 ± 5.39	44.77 ± 6.63	3.67 (2.21–5.12) *	12.10 (9.57–14.64) ‡	2.41
	IG (32)	47.66 ± 6.97	56.09 ± 5.28	−8.44 (−10.57–−6.30) **		
Total Lateral Flexion	CG (30)	62.30 ± 9.62	62.06 ± 9.54	0.24 (−1.37–1.84)	1.86 (−0.88–4.60)	0.34
	IG (32)	60.28 ± 9.86	61.91 ± 9.79	−1.62 (−3.90–0.65)		
Global Rotation	CG (30)	117.01 ± 18.36	113.23 ± 16.46	3.78 (1.28–6.25) *	18.39 (12.41–24.37) ‡	1.55
	IG (32)	111.56 ± 19.88	126.18 ± 16.33	−14.62 (−20.14–−9.11) **		
Shoulder ROM (degrees)						
Right Flexion	CG (30)	156.57 ± 15.75	154.83 ± 16.09	1.73 (−0.23–3.70)	10.67(7.21–14.13) ‡	1.56
	IG (32)	153.84 ± 14.93	162.78 ± 14.40	−8.93 (−11.85–−6.02) **		
Left Flexion	CG (30)	153.43 ± 15.03	153.07 ± 15.09	0.36 (−0.87–1.60)	10.70(7.84–13.57) ‡	1.89
	IG (32)	151.63 ± 12.06	161.97 ± 9.77	−10.34 (−12.96–−7.72) **		
Right Extension	CG (30)	32.3 ± 7.02	31.67 ± 6.64	0.63 (−0.58–1.84)	3.85 (2.07–5.64) ‡	1.09
	IG (32)	30.03 ± 6.64	33.25 ± 7.17	−3.22 (−4.58–−1.85) **		
Left Extension	CG (30)	29.00 ± 7.48	30.33 ± 7.70	−1.33 (−2.69–0.02)	0.01(−1.86–1.88)	0.28
	IG (32)	31.59 ± 6.53	32.94 ± 6.74	−1.34 (−2.68–−0.002) *		
Right Abduction	CG (30)	154.4 ± 16.67	155.13 ± 15.67	−0.73 (−2.37–0.91)	8.08 (4.58–11.58) ‡	1.16
	IG (32)	150.94 ± 16.23	159.75 ± 16.98	−8.81 (−11.96–−5.66) **		
Left Abduction	CG (30)	150.90 ± 15.9	148.60 ± 16.06	2.30 (1.13–3.47) *	12.39 (8.82–15.96) ‡	1.76
	IG (32)	147.78 ± 16.00)	157.87 ± 13.80	−10.09 (−13.50–−6.69) **		
Handgrip strength (kg/m^2^)	CG	41.43 ± 6.76	40.74 ± 6.88	0.69 (0.37–1.05) *	3.43 (4.21–2.65) ‡	2.23
	IG	39.92 ± 7.42	42.63 ± 7.67	−2.71 (−3.42–−2.01) **		
Right Hand (kg/m^2^)	CG	20.98 ± 3.40	20.61 ± 3.59	0.37 (0.13–0.61) *	2.00 (1.58–2.42) ‡	2.41
	IG	20.26 ± 4.04	21.90 ± 3.98	−1.63 (−1.99–−1.28) **		
Left Hand (kg/m^2^)	CG	20.47 ± 3.53	20.13 ± 3.50	0.34 (0.14–0.54) *	1.43 (0.91–1.94) ‡	1.40
	IG	19.65 ± 3.61	20.73 ± 3.87	−1.08 (−1.56–−0.60) **		

Note: CG: Control Group; IG: Intervention Group; Pre: values before intervention; Post: values after intervention; Intra-Dif: Intragroup differences in Pre and Post (95% confidence interval); Inter-Dif: Intergroup differences in Pre and Post (95% confidence interval); ES: Effect Size. * and ** intragroup significative differences (*p* < 0.05 y *p* < 0.01, respectively); † and ‡ intergroup significative differences (*p* < 0.05 and *p* < 0.01, respectively).

## Data Availability

The datasets generated and/or analyzed during the current study are available from the corresponding author on reasonable request.
